# Preoperative prediction of the need for arterial and central venous catheterization using machine learning techniques

**DOI:** 10.1038/s41598-022-16144-z

**Published:** 2022-07-13

**Authors:** Jungyo Suh, Sang-Wook Lee

**Affiliations:** 1 Department of Urology, Asan Medical Center, University of Ulsan College of Medicine, Seoul, Republic of Korea; 2 Department of Anesthesiology and Pain Medicine, Asan Medical Center, University of Ulsan College of Medicine, 88 Olympic-ro 43-gil, Songpa-gu, Seoul, 05505 Republic of Korea

**Keywords:** Health care, Medical research

## Abstract

Some surgical patients require an arterial or central venous catheterization intraoperatively. This decision relied solely on the experience of individual anesthesiologists; however, these decisions are not easy for clinicians who are in an emergency or inexperienced. Therefore, applying recent artificial intelligence techniques to automatically extractable data from electronic medical record (EMR) could create a very clinically useful model in this situation. This study aimed to develop a model that is easy to apply in real clinical settings by implementing a prediction model for the preoperative decision to insert an arterial and central venous catheter and that can be automatically linked to the EMR. We collected and retrospectively analyzed data from 66,522 patients, > 18 years of age, who underwent non-cardiac surgeries from March 2019 to April 2021 at the single tertiary medical center. Data included demographics, pre-operative laboratory tests, surgical information, and catheterization information. When compared with other machine learning methods, the DNN model showed the best predictive performance in terms of the area under receiver operating characteristic curve and area under the precision-recall curve. Operation code information accounted for the largest portion of the prediction. This can be applied to clinical fields using operation code and minimal preoperative clinical information.

## Introduction

Some surgical patients require invasive arterial catheterization; for example, when intraoperative continuous arterial pressure measurements, frequent arterial blood gas sampling, or blood laboratory tests are anticipated during surgery^1–4^. In addition, a central venous catheter (CVC) may be needed for the administration of inotropic agents or massive transfusion during surgery^5–9^. However, the preoperative decision on the necessity of an arterial catheter (AC) or CVC is typically made based on the subjective experience of the anesthesiologist according to the risk of surgery. The individual skill of the surgeon, surgical difficulty, and patient’s comorbidity will influence this decision. These invasive techniques are associated with various perioperative complications; therefore, they should be avoided where possible^9–16^. It is important to perform these procedures only when absolutely necessary and ensure that an appropriate level of anesthetic management is achieved. This decision is similar to the preoperative risk prediction of surgery and anesthesia, which is also related to the efficient allocation of medical resources. Moreover, it is a very important area for both healthcare providers and patients. However, this decision is not easy for clinicians who are in an emergency situation or inexperienced. In the past, this decision relied solely on the experience of individual anesthesiologists; however, these decisions can now utilize the artificial intelligence techniques, whose use has exploded in recent years compared to traditional statistical methods in medical fields^17,18^. This means that unskilled anesthesiologists and related medical personnel can easily obtain assistance in the preoperative decision making regarding the necessity of an invasive catheterization in each situation. In addition, experienced anesthesiologists can be assisted in clinical decision making using a predictive model of artificial intelligence. Hence, it can reduce the workload of the anesthesiologist.

Previous predictive models of artificial intelligence required too many variables that were not clinically important, and most of them required a lot of computing resources^18–21^. Therefore, this study aimed to develop a model that is easy to apply in real clinical settings by implementing a model that can be predicted based on minimal medical information automatically extracted data from electronic medical record (EMR). Furthermore, our study will enhance the explainability of predictive models by using explainable artificial intelligence (XAI) techniques. This will provided a basis for clinical decisions together^22^.

## Results

### Study population characteristics

Data from 66,522 patients who had undergone non-cardiac surgery at the tertiary academic medical center were collected for modeling in this study. Supplementary Table 1 shows some of the collected datasets as examples. In addition, Supplementary Table 2 summarizes the characteristics of the parameters used in machine learning models. Surgery with an AC was performed in 29.1% of patients. Surgery that required a CVC was performed in 7.9% of patients. Table 1 summarizes the data characteristics according to arterial line insertion. In surgeries with an arterial line, 99.5% of patients received general anesthesia. By contrast, in surgeries without an arterial line, 70.8% of patients received general anesthesia, which was more common in surgery with an AC (Table 1). This indicated that most surgeries with arterial line were performed under general anesthesia. It was extremely rare for surgeries with regional or neuro-axial anesthesia to have an arterial line. In addition, 27% of surgeries with an arterial line had CVCs, while only 0.04% had CVCs without an arterial line (Table 1). In other words, most surgeries with a CVC also included an AC.

**Table 1:** Study group characteristics with and without arterial catheterization.

	Total (*n* = 62,618)	Without A-line (*n* = 46,899)	With A-line (*n* = 15,719)	*P*-value
**Demographic data**				
Age, years	54.7 ± 15.9	52.7 ± 15.8	59.9 ± 14.3	< 0.001
Sex, female	36,737 (58.7%)	29,753 (63.4%)	6984 (44.4%)	< 0.001
Body-mass index, kg/m^2^	24.1 ± 3.8	24.2 ± 3.8	24.2 ± 3.7	0.093
**Preoperative laboratory results**				
White blood cell, 10^3^/μL	6.8 ± 2.6	6.5 ± 2.2	6.8 ± 2.4	< 0.001
Hemoglobin, g/dL	12.7 ± 1.9	12.9 ± 1.7	12.5 ± 1.9	< 0.001
Platelet, 10^3^/μL	242.5 ± 75.0	245.8 ± 68.9	239.2 ± 77.7	< 0.001
Sodium, mmol/L	139.8 ± 2.6	140.1 ± 2.3	140.0 ± 2.5	< 0.001
Potassium, mmol/L	4.3 ± 0.4	4.2 ± 0.3	4.3 ± 0.3	< 0.001
Chloride, mmol/L	103.7 ± 3.0	103.9 ± 2.6	103.6 ± 2.9	< 0.001
Calcium, mg/dL	9.2 ± 0.5	9.3 ± 0.4	9.2 ± 0.5	< 0.001
BUN, mg/dL	16.2 ± 10.7	14.5 ± 6.1	16.2 ± 7.6	< 0.001
Creatinine, mg/dL	1.0 ± 1.3	0.8 ± 0.6	0.9 ± 0.8	< 0.001
Albumin, g/dL	3.7 ± 0.5	3.8 ± 0.4	3.7 ± 0.5	< 0.001
AST, IU/L	25.2 ± 29.9	23.9 ± 19.9	26.9 ± 29.2	< 0.001
ALT, IU/L	22.7 ± 31.6	21.7 ± 29.1	25.2 ± 35.3	< 0.001
Glucose, mg/dL	114.9 ± 38.7	110.9 ± 34.5	121.7 ± 42.2	< 0.001
PT, INR	1.0 ± 0.1	1.0 ± 0.1	1.0 ± 0.1	< 0.001
aPTT, s	27.5 ± 3.7	27.2 ± 2.8	27.5 ± 3.8	< 0.001
**Type of anesthesia, *n***				
General anesthesia	48,871 (78.0%)	33,226 (70.8%)	15,645 (99.5%)	< 0.001
Neuro-axial anesthesia	2558 (4.1%)	2474 (5.3%)	84 (0.5%)	< 0.001
MAC	1459 (2.3%)	1378 (2.9%)	81 (0.5%)	< 0.001
Regional anesthesia	274 (0.4%)	269 (0.6%)	5 (0.03%)	< 0.001
Emergency surgery, n	5662 (9.0%)	5594 (11.9%)	68 (0.4%)	< 0.001
Central venous catheterization, n	4,365 (7.0%)	17 (0.04%)	4348 (27.0%)	< 0.001

Data represent mean ± standard deviation, median (interquartile range), or number (percentage).

A-line, arterial line; BUN, blood urea nitrogen; AST, aspartate aminotransferase; ALT, alanine aminotransferase; PT, prothrombin time; aPTT, Activated partial thromboplastin time; MAC, monitored anesthesia care.

### Missing data characteristics

Missing value characteristics are shown in Supplementary Table 3. The mean proportion of missing data in the whole dataset is 8.22%. The missing ratio of data for American Society of Anesthesiologists physical status (ASA-PS) class and the type of anesthesia was higher than other data. This high missing rate is related to the nature of human putting data directly into databases. Variables with high missing ratios showed no strong correlation with missing values of other variables (Supplementary Fig. 1). It can be seen that these missing values do not show any specific pattern, and these missing values are made randomly (Supplementary Fig. 2). Considering these random missing events, the missing values were replaced by the median values.

**Figure 1:**
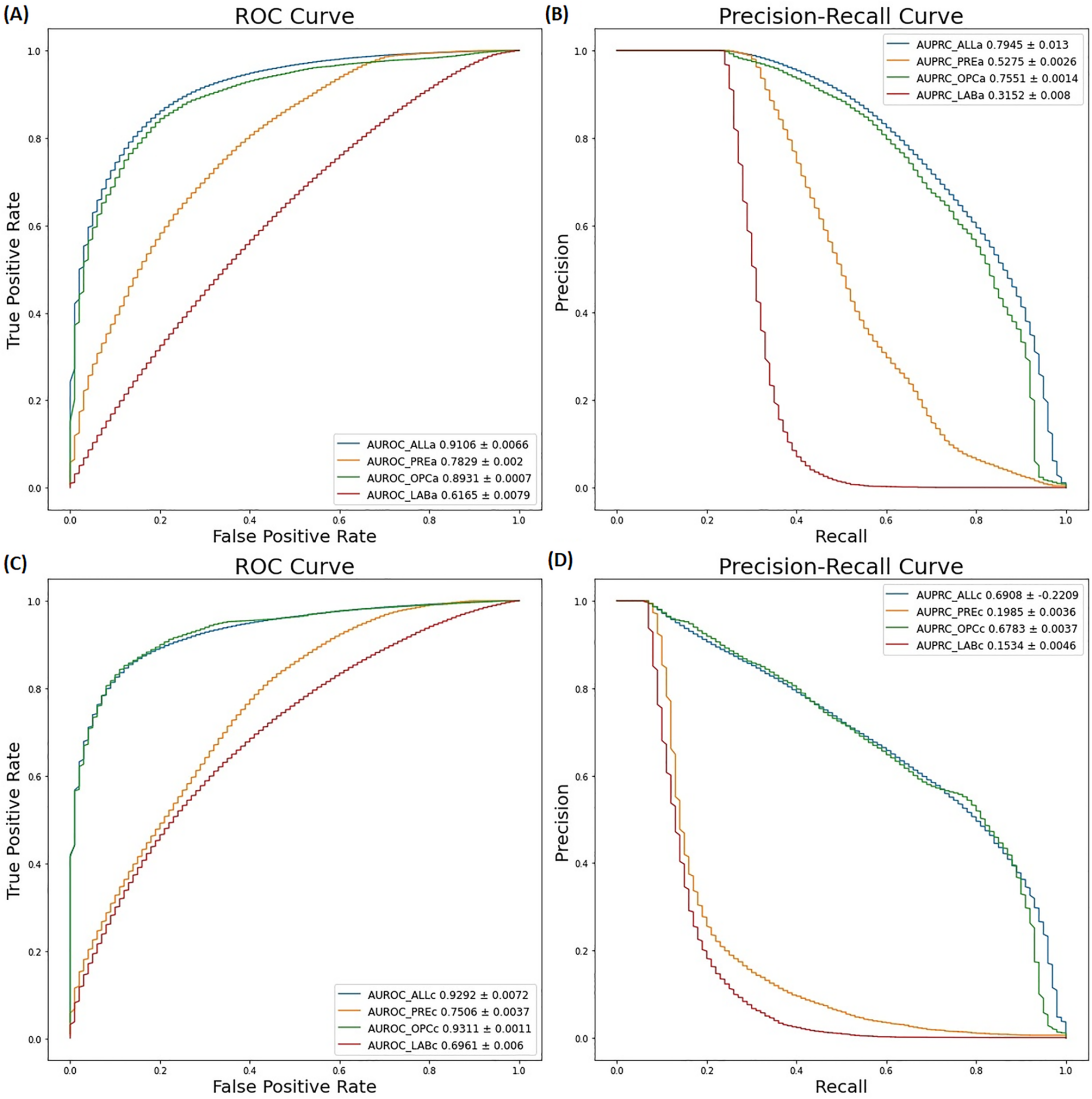
The predictive performance of the predictive models for several feature combinations using the deep learning method with 5 layers of deep neural network. (**A**) AUROC and (**B**) AUPRC of the predictive model for the preoperative decision on whether an arterial catheter is required during surgery. (**C**) AUROC and (**D**) AUPRC of the predictive model for the preoperative decision on whether a central venous catheter is required during surgery. AUROC and AUPRC values are represented as 95% confidence intervals. AUROC, area under receiver operating characteristic; AUPRC, area under precision-recall curve; DNN, deep neural network. ALLa, prediction for arterial catheterization using all variables; PREa, prediction for arterial catheterization using preoperative clinical data except for operation code and laboratory data; OPCa, prediction for arterial catheterization using operation codes; LABa, prediction for arterial catheterization using preoperative laboratory data; ALLc, prediction for central venous catheterization using all variables; PREc, prediction for central venous catheterization using preoperative clinical data except for operation code and laboratory data; OPCc, prediction for central venous catheterization using operation codes; LABc, prediction for central venous catheterization using preoperative laboratory data.

**Figure 2:**
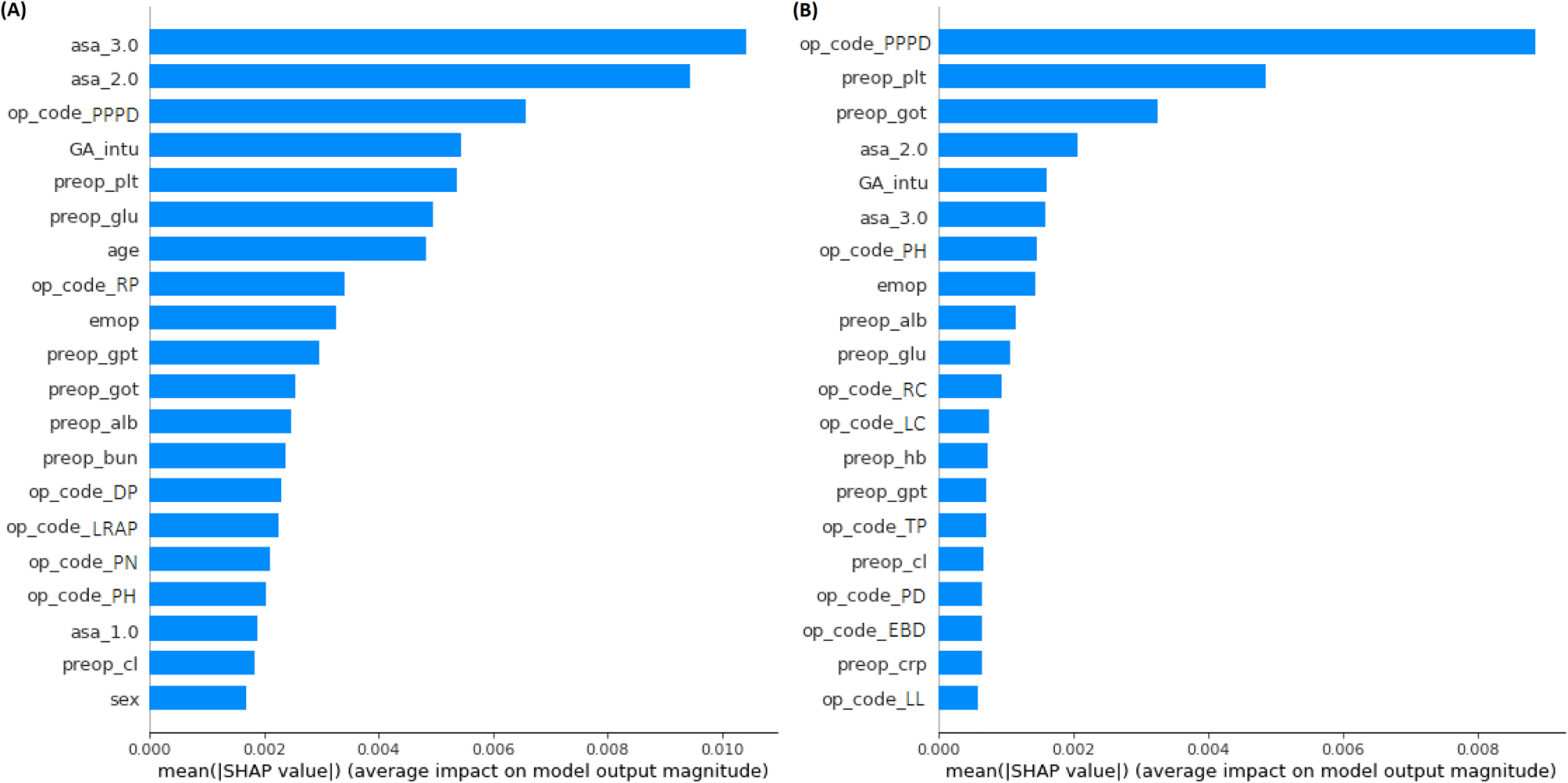
Feature importance of the DNN model for preoperative prediction for the necessity of invasive catheter insertion via SHAP assessment. (**A**) Feature importance of the DNN model for the preoperative prediction of the need for an arterial catheter insertion (**B**) Feature importance of the DNN model for the preoperative prediction of the need for a central venous catheter insertion. SHAP, SHapley Additive exPlanation; DNN, deep neural network; PPPD, pylorus preserving pancreaticoduodenectomy; GA_intu, general anesthesia with endotracheal intubation; plt, platelet; glu, glucose; RP, radical prostatectomy; emop, emergency operation; gpt, glutamate pyruvate transaminase; got, glutamate oxaloacetate transaminase; alb, albumin; bun, blood urea nitrogen; DP, distal pancreatectomy; LRAP, laparoscopic robotic assisted procedure; PN, partial nephrectomy; PH, partial hepatectomy; cl, chloride; RC, radical cystectomy; LC, laparoscopic cholecystectomy; hb, hemoglobin; TP, total pancreatectomy; PD, pancreaticoduodenectomy; EBD, excision of other bile duct; crp, c-reactive protein; LL, lobectomy of liver.

### Model performance results

The predictive performance of arterial catheterization according to each modeling method for the various combinations of features are shown in Table 2. In all data groups, the deep neural network (DNN) model had the best predictive performance when compared with other machine learning methods (Table 2). Supplementary Fig. 3 shows the train and validation learning curves according to the learning epoch of the DNN model. Although the learning curve fluctuates more in the test dataset than in the training dataset, the overall accuracy increases and the loss tends to decrease. According to predictive performance for the different selections of features, the mean area under the receiver operating characteristics (AUROC) value was 0.9089 when all features were used, followed by 0.893 when the operation code alone was used. By contrast, the mean AUROC value was 0.7835 when the preoperative data, such as preoperative demographic data and ASA-PS class, was used, followed by 0.605 when preoperative laboratory test finding were used. This showed a low predictive performance (Fig. 1).

**Table 2:** Predictive performance of arterial catheterization according to each modeling method using the deep or machine learning technique and a combination of features.

Features	Model	AUROC	AUPRC	F1-score
Preoperative data	DNN	0.7835 ± 0.0016	0.5296 ± 0.0020	0.3939 ± 0.0233
	XGBoost	0.6017 ± 0.0008	0.3385 ± 0.0014	0.3542 ± 0.0019
	RF	0.5947 ± 0.0012	0.3302 ± 0.0015	0.3368 ± 0.0031
	LR	0.5985 ± 0.0008	0.3358 ± 0.0012	0.3464 ± 0.0018
Laboratory data	DNN	0.6050 ± 0.0107	0.3061 ± 0.0093	0.0865 ± 0.0311
	XGBoost	0.5208 ± 0.0005	0.2555 ± 0.0010	0.0960 ± 0.0014
	RF	0.5196 ± 0.0010	0.2500 ± 0.0011	0.1106 ± 0.0037
	LR	0.5008 ± 0.0002	0.2367 ± 0.0008	0.0094 ± 0.0006
Operation code	DNN	0.8930 ± 0.0007	0.7548 ± 0.0015	0.6770 ± 0.0021
	XGBoost	0.6765 ± 0.0008	0.4641 ± 0.0017	0.5188 ± 0.0018
	RF	0.5288 ± 0.0049	0.2760 ± 0.0067	0.1095 ± 0.0175
	LR	0.7338 ± 0.0009	0.5293 ± 0.0017	0.6226 ± 0.0016
All features	DNN	0.9089 ± 0.0093	0.7943 ± 0.0118	0.4352 ± 0.0760
	XGBoost	0.7262 ± 0.0010	0.5292 ± 0.0017	0.6121 ± 0.0018
	RF	0.5444 ± 0.0050	0.2952 ± 0.0064	0.1659 ± 0.0169
	LR	0.6244 ± 0.0009	0.3547 ± 0.0014	0.4105 ± 0.0018

Data represent means (95% confidence intervals).

AUROC, area under receiver operating characteristic; AUPRC, area under precision-recall curve; DNN, deep neural network; XGBoost, extreme gradient boosting; DT, decision tree; RF, random forest; LR, logistic regression; ASA-PS, American Society of Anesthesiologists physical status.

**Figure 3:**
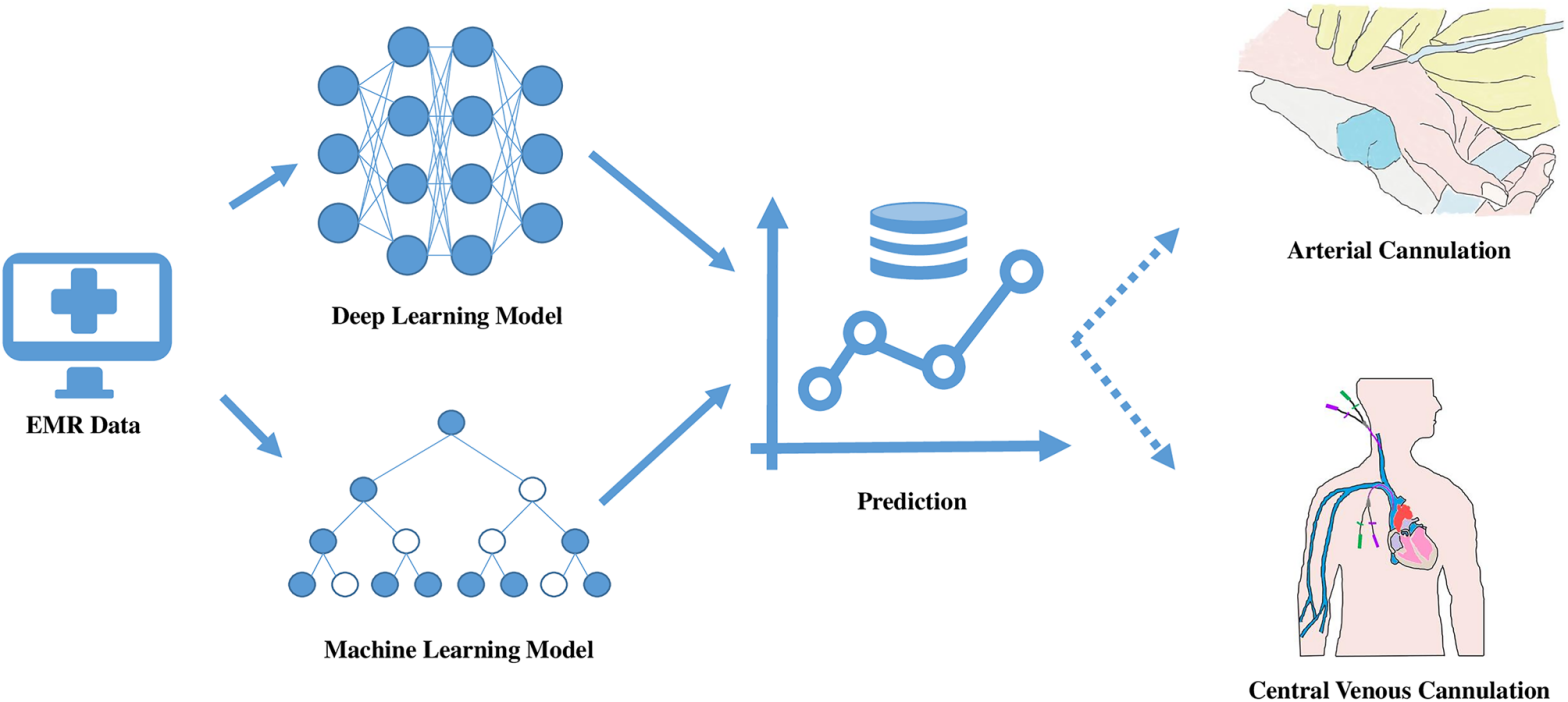
Schematic showing the development the predictive model for the preoperative decision of the necessity of arterial catheter or central venous catheter insertion during surgery.

Table 3 shows the predictive performance of preoperative predictions regarding the requirement of a CVC during surgery for each combination of features. Similar to the arterial catheterization results, the DNN model showed the best prediction performance when compared with the other machine learning methods. The AUROC value of the predictive model using operation code data alone was 0.9308, followed by an AUROC value of 0.9261 when all the input data were used. By contrast, models using all input variables performed better based on the area under the precision-recall curve (AUPRC) value; the predictive model using operation code information alone had an AUPRC value of 0.6754 compared with 0.6849 for the model using all input data. Figure 1 compares the predictive performance for AC and CVC of the DNN models in each dataset.

**Table 3:** Predictive performance of central venous catheterization according to each modeling method using deep learning or machine learning technique for the combination of various features.

Features	Model	AUROC	AUPRC	F1-score
Preoperative data	DNN	0.7527 ± 0.0022	0.2004 ± 0.0030	0.0499 ± 0.0091
	XGBoost	0.5131 ± 0.0005	0.0807 ± 0.0009	0.0520 ± 0.0017
	RF	0.5152 ± 0.0010	0.0792 ± 0.0014	0.0608 ± 0.0037
	LR	0.5156 ± 0.0006	0.0820 ± 0.0010	0.0620 ± 0.0023
Laboratory data	DNN	0.6966 ± 0.0054	0.1536 ± 0.0049	0.0026 ± 0.0017
	XGBoost	0.5154 ± 0.0006	0.0844 ± 0.0013	0.0608 ± 0.0025
	RF	0.5167 ± 0.0008	0.0762 ± 0.0011	0.0681 ± 0.0028
	LR	0.5016 ± 0.0001	0.0644 ± 0.0004	0.0080 ± 0.0007
Operation code	DNN	0.9308 ± 0.0012	0.6754 ± 0.0036	0.6400 ± 0.0055
	XGBoost	0.6673 ± 0.0016	0.3495 ± 0.0032	0.4918 ± 0.0036
	RF	0.5361 ± 0.0118	0.1279 ± 0.0212	0.1241 ± 0.0384
	LR	0.6257 ± 0.0014	0.2788 ± 0.0028	0.3962 ± 0.0036
All features	DNN	0.9261 ± 0.0097	0.6849 ± 0.0219	0.3687 ± 0.0658
	XGBoost	0.7062 ± 0.0015	0.4146 ± 0.0032	0.5699 ± 0.0032
	RF	0.5371 ± 0.0076	0.1283 ± 0.0137	0.1337 ± 0.0255
	LR	0.5057 ± 0.0004	0.0683 ± 0.0006	0.0248 ± 0.0016

Data are presented as means (95% confidence intervals).

AUROC, area under receiver operating characteristic; AUPRC, area under precision-recall curve; DNN, deep neural network; XGBoost, extreme gradient boosting; DT, decision tree; RF, random forest; LR, logistic regression; ASA-PS, American Society of Anesthesiologists physical status.

Supplementary Fig. 4 shows the predictive performance of the DNN model according to age and gender. The prediction of arterial catheter insertion was higher for men than for women, but the difference between men and women was not significant in the prediction of central venous catheter insertion. Furthermore, the predictive performance of the DNN model according to the age group shows the highest performance in the 60 s group and the tendency to decrease in the rest of the age group is shown in the prediction of all types of catheters insertion.

Supplementary Fig. 5 shows the receiver operating characteristics and precision-recall curves of 10 folds and mean values, respectively, by applying the tenfold cross-validation method in the performance evaluation of the extreme gradient boosting (XGBoost) model among prediction models for both type of catheters insertion.

Supplementary Fig. 6 shows calibration plots of various predictive modeling methods to evaluate the bias of predictions. Overall, the bias of the predictions predicted by the XGBoost model and the random forest (RF) model was smaller than those of other models.

### Feature importance

Figure 2 shows the feature importance in the deep learning model using the SHapley Additive exPlanation (SHAP) values. The most important feature of the preoperative prediction of the need for an AC insertion was ASA-PS class III. Interestingly, the most important feature of the preoperative prediction of the need for a CVC insertion was the operation code for pylorus preserving pancreatoduodectomy.

## Discussion

In this study, we identified the possibility of developing and applying a model that can predict whether preoperative invasive techniques are implemented using preoperative clinical data. Our results showed that the use of operation code information alone demonstrated a fairly accurate predictive performance. In addition, the DNN model showed the best predictive performance when compared with the other machine learning methods tested in this study.

AC cannulation often causes complications, such as cannula site pain, infection, bleeding, thrombus formation, ischemic injury, and nerve damage. CVC insertion also can cause complications, such as vascular injury, pneumothorax, arrhythmia, device dysfunction, and infection^1,9,16,23,24^. Therefore, these invasive procedures should only be performed when necessary during surgery^25^. By contrast, if these catheters are not secured before surgery, it can be difficult to deal with sudden hemodynamically unstable situations. If rapid transfusion is required due to unexpected massive bleeding during surgery, it can be very difficult to insert a CVC intraoperatively and these situations may delay the response to emergency situations. Therefore, it is very crucial to know before surgery whether the insertion of AC or CVC is needed during surgery. This decision is not made with any special principle, but rather requires a comprehensive consideration of operation risk, surgeon proficiency, and patient risk. In fact, this type of prediction is closely related to preoperative risk prediction. In the past, most of these decisions are finalized based on the personal experience of anesthesiologist; however, it was hypothesized that a better prediction model could be created by applying machine learning techniques based on data.

Although it is not difficult to make these kinds of decisions before surgery for skilled anesthesiologists, inexperienced trainees and other medical assistants may find it difficult to take these decisions before surgery. Therefore, a preoperative prediction for invasive catheterization could help these less experienced staff members make clinical decisions. Furthermore, it can reduce the workload of the experienced anesthesiologist to receive help from the automatic decision system of the predictive model.

In our study, operation code information is the most important part of the prediction model. We included 1,257 operation codes, which were one-hot encoded, as each input variable to perform the predictive modeling. In the case of predictions with so many input variables, it would have been impossible to directly enter individual operation codes using classical statistical methods. One statistical rule of thumb states that the dataset should be at least 10 times the number of input variables^26^. Therefore, at least 12,570 data points would be required in this study when modeling in a statistical manner with minimum operation code information alone. Although modeling could be possible in a statistical method if more than 60,000 patients enrolled in our study, the development of predictive models using the full operation code information is very limited because the reality of clinical research means that it is difficult to enroll more than 10,000 people per study. In previous studies, predictive models are usually developed by dividing operation codes into several groups^27,28^. However, if machine learning techniques are used in modeling, all the operation code information can be inputted. Hence, a predictive model can be developed without grouping many operation codes.

In this study, the DNN model showed the best predictive performance when compared with the other machine learning techniques. In previous studies, ensemble models and classical machine learning methods have shown little or no difference in predictive performance when compared with the DNN model^29^. However, when the number of input variables increases as in this study, it can be seen that the DNN model shows better predictive performance than the conventional machine learning techniques. Hence, this study shows that the DNN model has superior predictive power when considering predictions with many features.

We used a bootstrap method in model performance validation. This method is to understand machine learning performance in a conventional approach. Presenting a confidence interval for evaluating performance using bootstrap is a preferred method for many conventional researchers because it is proposed in a more traditional way to understand the performance of the model. In addition, the use of this bootstrap method for machine learning is one of the important techniques that can prevent overfitting in learning the imbalanced data.

The strength of our study is that this study is the first to implement a model that predicts preoperative decision making for intraoperative invasive techniques using operation code information and minimal clinical information before surgery, to the best of our knowledge. This decision support tool using information that can be automatically extracted from the EMR system is very useful in actual clinical situations. Therefore, our study can be said to be a cornerstone in that it presents a model applicable to the actual clinical field by linking this automated decision support tool with the EMR system. In addition, unlike conventional risk prediction models, this model can perform predictions by using the full operation code information. Another strength of this study is that the explanatory power of the predictive model was increased using XAI techniques.

One limitation of our study is that it is performed in a single center. Therefore, it is difficult to apply the predictive model developed in this study to other institutions. This is because operation code data contain information based on the characteristics of an individual hospital’s own system and this can affect the outcome. Therefore, the same operation code may have different parameters in each hospital. It may be difficult to apply the prediction model of this study to other institutions; however, it is possible to develop a prediction model that fits well with each institution by applying the same method of development used in this study with individual hospital data. Nonetheless, it will be necessary to test this model in a multi-center study in the future. In addition, since this study is based on a database collected retrospectively, it seems necessary to validate the performance of the model in future prospective studies. Another limitation of this study is that it did not reveal the clinical usefulness of this predictive model. It is not known how helpful these predictive models are to trainees or other medical assistants when applied in the clinical field. Evaluating whether the introduction of such a predictive model significantly reduces the sudden implementation of arterial or central venous catheterization intraoperatively or saves preoperative preparation time will indirectly show how helpful these predictive models are in clinical practice. Therefore, studies to evaluate the clinical usefulness of these predictive models should be conducted in the future.

In conclusion, we evaluated whether a predictive model for the preoperative decision to insert an AC and CVC could be developed and applied to clinical data using operation code and minimal preoperative clinical information automatically extracted from EMR. Therefore, if this prediction model can be automatically linked with the EMR system to help clinical decision, it will play a very important role as a practical decision support tool for emergency or inexperienced medical personnel. In the future, an applicable predictive model for different clinical situations should be conducted to confirm these data. In particular, multicenter studies on predictive models for the intraoperative placement of AC and CVC and its clinical usefulness should be conducted.

## Methods

### Study design and patients

This study was approved by the institutional review board (IRB) of the tertiary-care academic medical center (IRB No. 2021-1131). Written informed consent was exempted by the IRB because the research was conducted retrospectively. We conducted the study in accordance with the guidelines entitled “Guidelines for Development and Reporting Machine Learning Predictive Models in Biomedical Research: A Multidisciplinary View”^30^. All methods of our study were performed according to related guidelines and regulations. We collected and retrospectively analyzed data from patients > 18 years of age who underwent non-cardiac surgeries from March 2019 to April 2021 at the tertiary-care medical center. The exclusion criteria of our study were: patients who underwent heart surgeries, organ transplant surgeries, and neurosurgeries; and patients without information about catheterization during surgery.

### Preparing data for modeling

The data consisted of patient demographics, pre-operative laboratory tests, surgical information, and intra-operative catheterization information extracted from the hospital’s electronic medical record system. Demographic data included age, sex, height, weight, and body mass index. Preoperative laboratory tests included white blood cell, hemoglobin, and platelet counts, prothrombin time, activated partial thromboplastin time), sodium, potassium, chloride, calcium, blood urea nitrogen, creatinine, aspartate aminotransferase, alanine aminotransferase, albumin, glucose, and c-reactive protein concentrations. Surgical information included the emergency status of surgery, operation code, and type of anesthesia (general or regional). Additionally, we collected the ASA-PS class for each surgical patient evaluated preoperatively. The primary outcome was the implementation of intra-operative catheterization during surgery (Fig. 3). This was extracted as binary data based on the anesthesia records in the electronic medical records.

### Model building

Missing values in the input variables of the model were filled with the median values of each variable. For model variables to be used as inputs, all continuous variables were scaled and categorical variables were one-hot encoded^31^. The standard scaler function provided by the Scikit-Learn package was used to correct the range of various values of the model parameters. Through one-hot coding, 1,257 operation codes were entered into the model. Thus, 1,257 features were generated to create input variables for the model. For the prediction algorithm, conventional machine-learning methods, such as logistic regression algorithms, were used. Ensemble algorithms, such as RF and XGBoost, and deep learning methods, such as the DNN, were used to compare predictive performance^32–34^. The whole dataset was divided into training, validation, and test sets at a 6:2:2 ratio. The DNN model was a simple model consisting of five hidden layers. The basic structure of the model consisted of a stack of single layers of DNNs with a dropout rate of 0.5 after batch normalization and rectified linear unit activation functions were applied to the dense layer. Sigmoid activation functions were applied to the final output layer^35^. The learning rate for the training model was 0.001. Binary cross entropy was used as the loss function of the model. In addition, the Adaptive Moment Estimation optimizer was used^36^. The bootstrap method was used to measure the average performance of each predictive model^37^. By iterating the process of resampling training data several times using the bootstrap method, the predictive performance values of the models learned from multiple training datasets were expressed as mean values and confidence intervals. The bootstrap method was used because it can overcome problems of overfitting to a particular data distribution in learning imbalanced data. In addition, we used the tenfold cross validation method, which is one of the model performance evaluation methods, as a means of selecting a better model without bias on one side.

### Model evaluation

The predictive performance of the models was compared for the various combinations of features. We compared the predictive performances of (1) the model developed using only preoperative data, including demographic data and ASA class information; (2) the model developed using preoperative laboratory data alone; (3) the model developed from operational code data alone; and (4) the model developed from all the data. This comparison was used to determine the extent to which each part of the data had an impact on prediction. In addition, we used the SHAP values to analyze the feature importance of the predictive model^38^. We used the SHAP values to extract the important variables that had a significant impact on the outcome prediction. The predictive performance of each model was evaluated by comparing the AUROC, AUPRC, and F1 score. Additionally, various modeling methods were compared using a calibration plot to evaluate whether the predicted value was biased compared to the actual value.

### Statistical analysis and modeling tools

Continuous variables were represented by means and standard deviations. Categorical variables were represented by numbers and percentages. When comparing two groups of continuous variables, the *t*-test was used. The Chi-squared test is used to compare categorical variables. In this study, variables with *p*-values < 0.05 were considered statistically significant. Machine learning and deep learning algorithms were implemented using python 3.9 with the Scikit-Learn and TensorFlow packages.

## Supplementary Information

Supplementary Information.

## Data Availability

The datasets used during the current study are available from the corresponding author on reasonable request.

## Abbreviations

AC: Arterial catheter
aPTT: Activated partial thromboplastin time
ASA-PS: American Society of Anesthesiologists physical status
AUPRC: Area under the precision-recall curve
AUROC: Area under the receiver operating characteristics curve
BUN: Blood urea nitrogen
CRP: C-reactive protein
CVC: Central venous catheter
DNN: Deep neural network
IRB: Institutional review board
LR: Logistic regression
PT: Prothrombin time
RF: Random forest
SHAP: Shapley additive explanation
XAI: Explainable artificial intelligence
XGBoost: Extreme gradient boosting

## References

[1] Wilkins RG (1985). Radial artery cannulation and ischaemic damage: A review. Anaesthesia.

[2] Clark VL, Kruse JA (1992). Arterial catheterization. Crit. Care Clin..

[3] Cousins TR, O'Donnell JM (2004). Arterial cannulation: A critical review. AANA J..

[4] Brzezinski M, Luisetti T, London MJ (2009). Radial artery cannulation: A comprehensive review of recent anatomic and physiologic investigations. Anesth. Analg..

[5] Infusion Nurses S (2006). Infusion nursing standards of practice. J. Infus. Nurs..

[6] American Society of Anesthesiologists Task Force on Central Venous A (2012). Practice guidelines for central venous access: a report by the American Society of Anesthesiologists Task Force on Central Venous Access. Anesthesiology.

[7] Freel AC (2008). American College of Surgeons Guidelines Program: a process for using existing guidelines to generate best practice recommendations for central venous access. J. Am. Coll. Surg..

[8] Bodenham Chair A (2016). Association of anaesthetists of Great Britain and Ireland: Safe vascular access 2016. Anaesthesia.

[9] Smith RN, Nolan JP (2013). Central venous catheters. BMJ.

[10] Pittet D, Tarara D, Wenzel RP (1994). Nosocomial bloodstream infection in critically ill patients. Excess length of stay, extra costs, and attributable mortality. JAMA.

[11] Dezfulian C, Lavelle J, Nallamothu BK, Kaufman SR, Saint S (2003). Rates of infection for single-lumen versus multilumen central venous catheters: A meta-analysis. Crit. Care Med..

[12] Cook D (1997). Central venous catheter replacement strategies: a systematic review of the literature. Crit. Care Med..

[13] Pronovost P (2006). An intervention to decrease catheter-related bloodstream infections in the ICU. N. Engl. J. Med..

[14] Rooden CJ, Tesselaar ME, Osanto S, Rosendaal FR, Huisman MV (2005). Deep vein thrombosis associated with central venous catheters - A review. J. Thromb. Haemost..

[15] Kirkpatrick A, Rathbun S, Whitsett T, Raskob G (2007). Prevention of central venous catheter-associated thrombosis: A meta-analysis. Am. J. Med..

[16] Pikwer A, Akeson J, Lindgren S (2012). Complications associated with peripheral or central routes for central venous cannulation. Anaesthesia.

[17] Chang V, Bailey J, Xu QA, Sun Z (2022). Pima Indians diabetes mellitus classification based on machine learning (ML) algorithms. Neural Comput. Appl..

[18] Chiew CJ, Liu N, Wong TH, Sim YE, Abdullah HR (2020). Utilizing machine learning methods for preoperative prediction of postsurgical mortality and intensive care unit admission. Ann. Surg..

[19] Hill BL (2019). An automated machine learning-based model predicts postoperative mortality using readily-extractable preoperative electronic health record data. Br. J. Anaesth..

[20] Lee CK, Hofer I, Gabel E, Baldi P, Cannesson M (2018). Development and validation of a deep neural network model for prediction of postoperative in-hospital mortality. Anesthesiology.

[21] Seki T, Kawazoe Y, Ohe K (2021). Machine learning-based prediction of in-hospital mortality using admission laboratory data: A retrospective, single-site study using electronic health record data. PLoS ONE.

[22] Linardatos P, Papastefanopoulos V, Kotsiantis S (2021). Explainable AI: A review of machine learning interpretability methods. Entropy-Switz.

[23] Scheer B, Perel A, Pfeiffer UJ (2002). Clinical review: Complications and risk factors of peripheral arterial catheters used for haemodynamic monitoring in anaesthesia and intensive care medicine. Crit. Care.

[24] Kornbau C, Lee KC, Hughes GD, Firstenberg MS (2015). Central line complications. Int. J. Crit. Illn. Inj. Sci..

[25] Uemura K, Inoue S, Kawaguchi M (2018). The unnecessary application of central venous catheterization in surgical patients. Braz. J. Anesthesiol..

[26] Van Belle G (2002). Statistical Rules of Thumb.

[27] Shinall MC (2020). Association of preoperative patient frailty and operative stress with postoperative mortality. JAMA Surg..

[28] Lee SW (2021). Predictive model for the assessment of preoperative frailty risk in the elderly. J. Clin. Med..

[29] Park DJ (2021). Development of machine learning model for diagnostic disease prediction based on laboratory tests. Sci. Rep..

[30] Luo W (2016). Guidelines for developing and reporting machine learning predictive models in biomedical research: A multidisciplinary view. J. Med. Internet Res..

[31] Pedregosa FVG, Gramfort A (2011). Scikit-learn: Machine learning in Python. J. Mach. Learn. Res..

[32] LeCun Y, Bengio Y, Hinton G (2015). Deep learning. Nature.

[33] Breiman L (2001). Random forests. Mach. Learn..

[34] Balas, V. E., Borah, S., Kalita, J. & Pradhan, R. in *Advances in Intelligent Systems and Computing,* 1 online resource (XIV, 530 pages 233 illustrations) (Springer Singapore : Imprint: Springer,, Singapore, 2019).

[35] Agarap, A. F. Deep Learning using Rectified Linear Units (ReLU). *Computer Science, Mathematics ArXiv* (2018).

[36] Zhilu Zhang, M. R. S. Generalized Cross Entropy Loss for Training Deep Neural Networks with Noisy Labels. In *32nd Conference on Neural Information Processing Systems (NeurIPS 2018)* (2018). PMC11747755 39839708

[37] Boos DD (2003). Introduction to the bootstrap world. Stat. Sci..

[38] Lundberg SM, L. S.-I. A Unified Approach to Interpreting Model Predictions., 4765–4774 (2017).

