# Supplementary material for: Preoperative prediction of the need for arterial and central venous catheterization using machine learning techniques
Source: Sci Rep. 2022 Jul 13;12:11948. doi: 10.1038/s41598-022-16144-z (PMC9279292; doi:10.1038/s41598-022-16144-z)
Supplement: Supplementary file 1 — Supplementary Information. [file 41598_2022_16144_MOESM1_ESM.docx]

**Supplementary information**

**Preoperative prediction of the need for arterial and central venous catheterization using machine learning techniques**

Jungyo Suh MD^1^, Sang-Wook Lee MD PhD^2^*.

1. Department of Urology, Asan Medical Center, University of Ulsan College of Medicine, Seoul, Republic of Korea

2. Department of Anesthesiology and Pain Medicine, Asan Medical Center, University of Ulsan College of Medicine, Seoul, Republic of Korea

**Contents**

**Supplementary Table 1.** Sample dataset.

**Supplementary Table 2.** Parameters of the machine learning models.

**Supplementary Table 3.** Missing value characteristics.

**Supplementary Figure 1.** Nullity correlation heatmap of missing data.

**Supplementary Figure 2.** Distribution of missing values and data completeness of dataset.

**Supplementary Figure 3.(a)** Train and validation learning curve of DNN model for arterial catheterization.

**Supplementary Figure 3.(b)** Train and validation learning curve of DNN model for central venous catheterization.

**Supplementary Figure 4.(a)** Prediction performance of DNN model for arterial catheterization by gender using all features.

**Supplementary Figure 4.(b)** Prediction performance of DNN model for arterial catheterization by age groups using all features.

**Supplementary Figure 4.(c)** Prediction performance of DNN model for central venous catheterization by gender using all features.

**Supplementary Figure 4.(d)** Prediction performance of DNN model for central venous catheterization by age groups using all features.

**Supplementary Figure 5.(a)** ROC and PR curves of XGB model with 10-fold cross-validation for predictive performance of arterial catheterization using all features.

**Supplementary Figure 5.(b)** ROC and PR curves of XGB model with 10-fold cross-validation for predictive performance of central venous catheterization using all features.

**Supplementary Figure 6.(a)** Calibration curve of predictive performance for arterial catheterization according to various modeling methods using all features.

**Supplementary Figure 6.(b)** Calibration curve of predictive performance for central venous catheterization according to various modeling methods using all features.

**Supplementary Table 1.** Sample dataset

| ID | | Pt1 | Pt2 | Pt3 | Pt4 |  | Pt67609 | Pt67610 |
| --- | --- | --- | --- | --- | --- | --- | --- | --- |
| Demographic data | Age | 66 | 37 | 67 | 59 |  | 53 | 57 |
|  | Sex | 0 | 1 | 0 | 1 |  | 0 | 1 |
|  | BMI | 25.1 | 21.1 | 30.4 | 27.7 |  | 22.1 | 23.9 |
|  | Emop | 1 | 0 | 0 | 1 |  | 0 | 0 |
|  | ASA | NULL | 1 | NULL | NULL |  | 2 | NULL |
| Laboratory data | WBC | 4.27 | 4.60 | 14.72 | 4.95 |  | 8.00 | 4.30 |
|  | Plt | 133.0 | 222.0 | 137.9 | 174.4 |  | 167.0 | 177.0 |
|  | Hb | 12.5 | 12.8 | 9.5 | 12.8 |  | 12.7 | 13.4 |
|  | Na | 139.7 | 141.0 | 141.9 | 142.7 |  | 141.0 | 141.0 |
|  | K | 4.3 | 4.2 | 4.1 | 4.2 |  | 3.9 | 4.1 |
|  | Cl | 105.0 | 102.0 | 108.1 | 104.3 |  | 107.0 | 108.0 |
|  | Ca | 8.0 | 10.0 | 7.6 | 9.4 |  | 9.6 | 8.5 |
|  | BUN | 14.7 | 9.0 | 20.2 | 12.3 | **……** | 13.0 | 15.0 |
|  | Cr | 1.02 | 0.53 | 0.82 | 0.60 |  | 0.94 | 0.70 |
|  | Alb | 3.2 | 4.4 | 2.6 | 3.7 |  | 3.6 | 3.9 |
|  | Glu | 93 | 93 | 192 | 116 |  | 106 | 104 |
|  | AST | 29.3 | 22.0 | 28.5 | 15.3 |  | 49.0 | 28.0 |
|  | ALT | 20.0 | 23.0 | 25.5 | 14.0 |  | 18.5 | 21.0 |
|  | PT | 0.99 | 1.02 | 1.22 | 0.97 |  | 1.11 | 1.02 |
|  | aPTT | 27.0 | 24.8 | 27.7 | 26.3 |  | 24.6 | 25.4 |
| Anesthesia type | SA | 0 | 0 | 0 | 0 |  | 0 | 0 |
|  | EA | 0 | 0 | 0 | 0 |  | 0 | 0 |
|  | CSE | 0 | 0 | 0 | 0 |  | 0 | 0 |
|  | BPB | 0 | 0 | 0 | 0 |  | 0 | 0 |
|  | GA_intu | 1 | 1 | 1 | 1 |  | 1 | 1 |
|  | GA_mask | 0 | 0 | 0 | 0 |  | 0 | 0 |
| Operation code | OC_1742 | 1 | 0 | 0 | 0 |  | 0 | 0 |
|  | OC_4603 | 0 | 0 | 0 | 0 |  | 1 | 0 |
|  | **…** |  |  |  |  |  |  |  |
|  | OC_8339 | 0 | 0 | 0 | 1 |  | 0 | 0 |
|  | OC_8595 | 0 | 1 | 0 | 0 |  | 0 | 0 |
| Outcome data | A-line | 0 | 0 | 1 | 0 |  | 0 | 0 |
|  | C-line | 0 | 0 | 0 | 0 |  | 0 | 0 |

Pt, Patient; BMI, Body mass index; Emop, Emergency operation; ASA-PS, American society of anesthesiologists physical status; WBC, White blood cells; Plt, Platelet; Hb, Hemoglobin; Na, Natrium; K, Kalium; Cl, Chloride; Ca, Calcium; BUN, Blood urea nitrogen; Cr, Creatinine; Alb, Albumin; Glu, Glucose; AST, Aspartate aminotransferase; ALT, Alanine aminotransferase; PT, Prothrombin time; aPTT, Activated partial thromboplastin time; SA, Spinal anaesthesia; EA, Epidural anesthesia; CSE, Combined spinal-epidural anaesthesia; BPB, Brachial plexus block; GA_intu, General anaesthesia with intubation; GA_mask, General anaesthesia with mask bagging; OC, Operation code; A-line, Arterial line; C-line, Central line.

**Supplementary Table 2.** Parameters of the machine learning models.

| Parameters | Description | Data type | Range |
| --- | --- | --- | --- |
| Demographic data |  |  |  |
| Age | Age (years) | Numeric | [18, 100] |
| Sex | Female = 1, Male =0. | Factor | [0, 1] |
| BMI | Body mass index (kg/m^2^) | Numeric | [5.7, 49.8] |
| Surgical Informations |  |  |  |
| emop | Emergency status of surgery | Factor | [0, 1] |
| ASA | American society of anesthesiologists physical status | Factor | [1, 6] |
| Type of Anesthesia |  |  |  |
| GA_intu | General anesthesia with intubation | Factor | [0, 1] |
| GA_mask | General anesthesia with mask bagging | Factor | [0, 1] |
| SA | Spinal anesthesia | Factor | [0, 1] |
| EA | Epidural anesthesia | Factor | [0, 1] |
| BPB | Brachial plexus block | Factor | [0, 1] |
| CSE | Combined spinal-epidural anesthesia | Factor | [0, 1] |
| Laboratory data |  |  |  |
| WBC | White blood cells | Numeric | [0.02, 119.5] |
| Plt | Platelet | Numeric | [7.3, 1085.0] |
| Hb | Hemoglobin | Numeric | [4.5, 20.8] |
| Na | Sodium | Numeric | [113.0, 155.4] |
| K | Potassium | Numeric | [2.4, 7.5] |
| Cl | Chloride | Numeric | [72.6, 121.0] |
| Ca | Calcium | Numeric | [4.3, 18.0] |
| AST | Aspartate aminotransferase | Numeric | [5.0, 3157.7] |
| ALT | Alanine aminotransferase | Numeric | [4.0, 1055.0] |
| Alb | Albumin | Numeric | [0.7, 5.4] |
| BUN | Blood urea nitrogen | Numeric | [2.0, 238.1] |
| Cr | Creatinine | Numeric | [0.17, 22.95] |
| Glu | Glucose | Numeric | [35, 666] |
| PT | Prothrombin time | Numeric | [0.69, 8.56] |
| aPTT | Activated partial thromboplastin time | Numeric | [19, 180] |
| Operation code | 1257 operation codes | Factor | [0, 1] |

**Supplementary Table 3.** Missing value characteristics

| Characteristics | Available Data | Missing data | Missing Data (%) |  |
| --- | --- | --- | --- | --- |
| Demographics |  |  |  |  |
| Age | 67,607 | 3 | 0.00 |  |
| Sex | 67,607 | 3 | 0.00 |  |
| Body Mass Index | 61,877 | 5753 | 8.51 |  |
| Preoperative Laboratory Tests |  |  |  |  |
| White Blood Cells | 65,832 | 1778 | 2.63 |  |
| Hemoglobin | 65,832 | 1778 | 2.63 |  |
| Platelet | 65,832 | 1778 | 2.63 |  |
| Sodium | 64,451 | 3159 | 4.67 |  |
| Potassium | 64,451 | 3159 | 4.67 |  |
| Chloride | 64,311 | 3299 | 4.88 |  |
| Calcium | 64,913 | 2697 | 3.99 |  |
| Blood Urea Nitrogen | 62,487 | 5123 | 7.58 |  |
| Creatinine | 65,461 | 2149 | 3.18 |  |
| Albumin | 65,805 | 1805 | 2.67 |  |
| Aspartate aminotransferase | 65,847 | 1763 | 2.61 |  |
| Alanine aminotransferase | 65,847 | 1763 | 2.61 |  |
| Glucose | 65,427 | 2183 | 3.23 |  |
| Prothrombin Time | 61,795 | 5815 | 8.60 |  |
| Activated Partial Thromboplastin Time | 61,389 | 6221 | 9.20 |  |
| Surgical Informations |  |  |  |  |
| Emergency status of surgery | 67,607 | 3 | 0.00 |  |
| ASA-PS Class | 59,815 | 7795 | 11.53 |  |
| Type of Anesthesia |  |  |  |  |
| EA | 55,406 | 12,204 | 18.05 |  |
| SA | 55,406 | 12,204 | 18.05 |  |
| CSE | 55,406 | 12,204 | 18.05 |  |
| BPB | 55,406 | 12,204 | 18.05 |  |
| GA_intu | 55,406 | 12,204 | 18.05 |  |
| GA_mask | 55,406 | 12,204 | 18.05 |  |
| Catheterization |  |  |  |  |
| Arterial catheterization | 55,406 | 12,204 | 18.05 |  |
| Central venous catheterization | 55,406 | 12,204 | 18.05 |  |
| Mean of missing data (%) |  |  | 8.22 |  |

ASA-PS, American society of anesthesiologists physical status; EA, Epidural anesthesia; SA, Spinal anaesthesia; CSE, Combined spinal-epidural anaesthesia; BPB, Brachial plexus block; GA_intu, General anaesthesia with intubation; GA_mask, General anaesthesia with mask bagging.

**Supplementary Figure 1.** Nullity correlation heatmap of missing data.


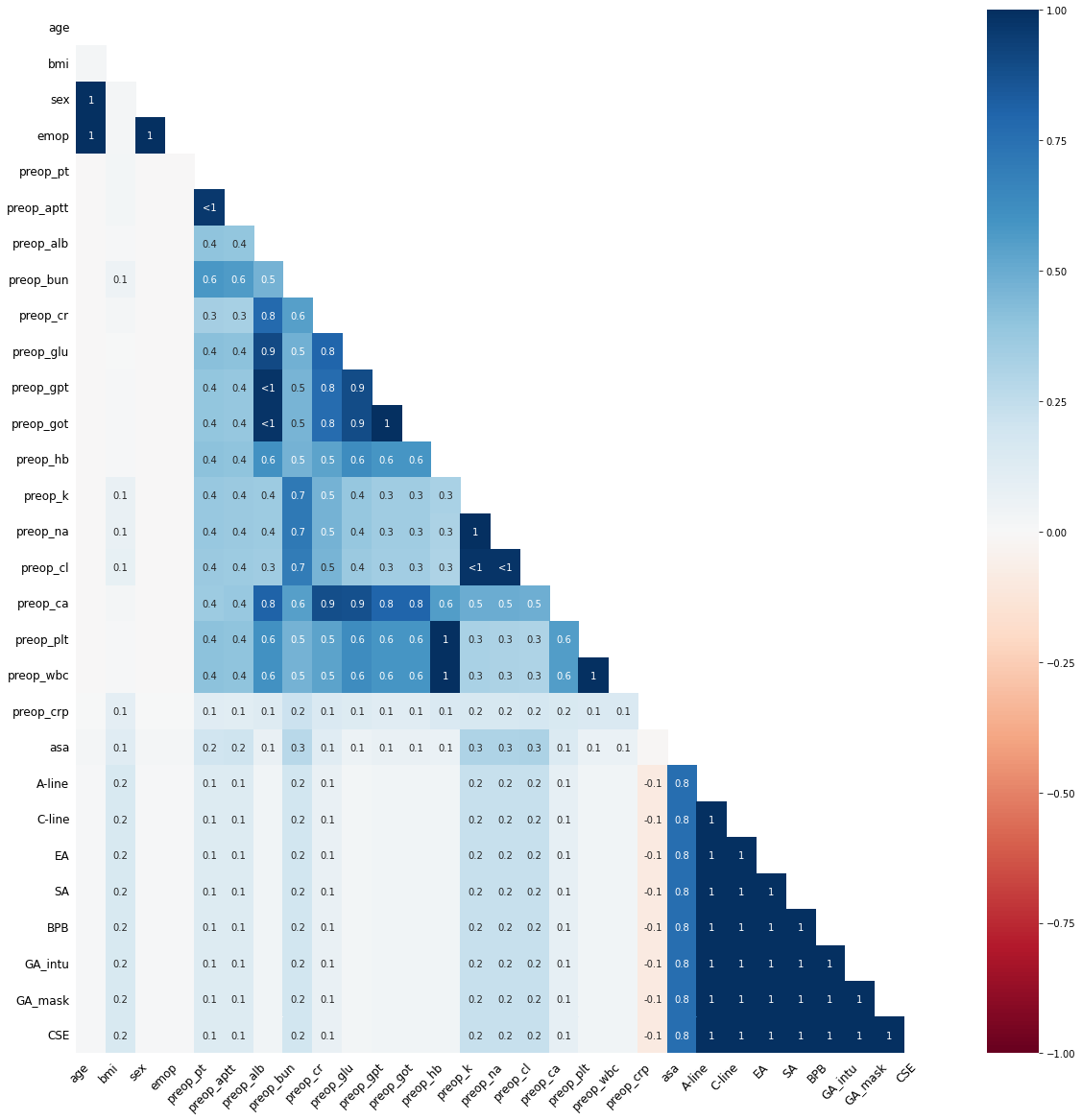


**Supplementary Figure 2.** Distribution of missing values and data completeness of dataset.


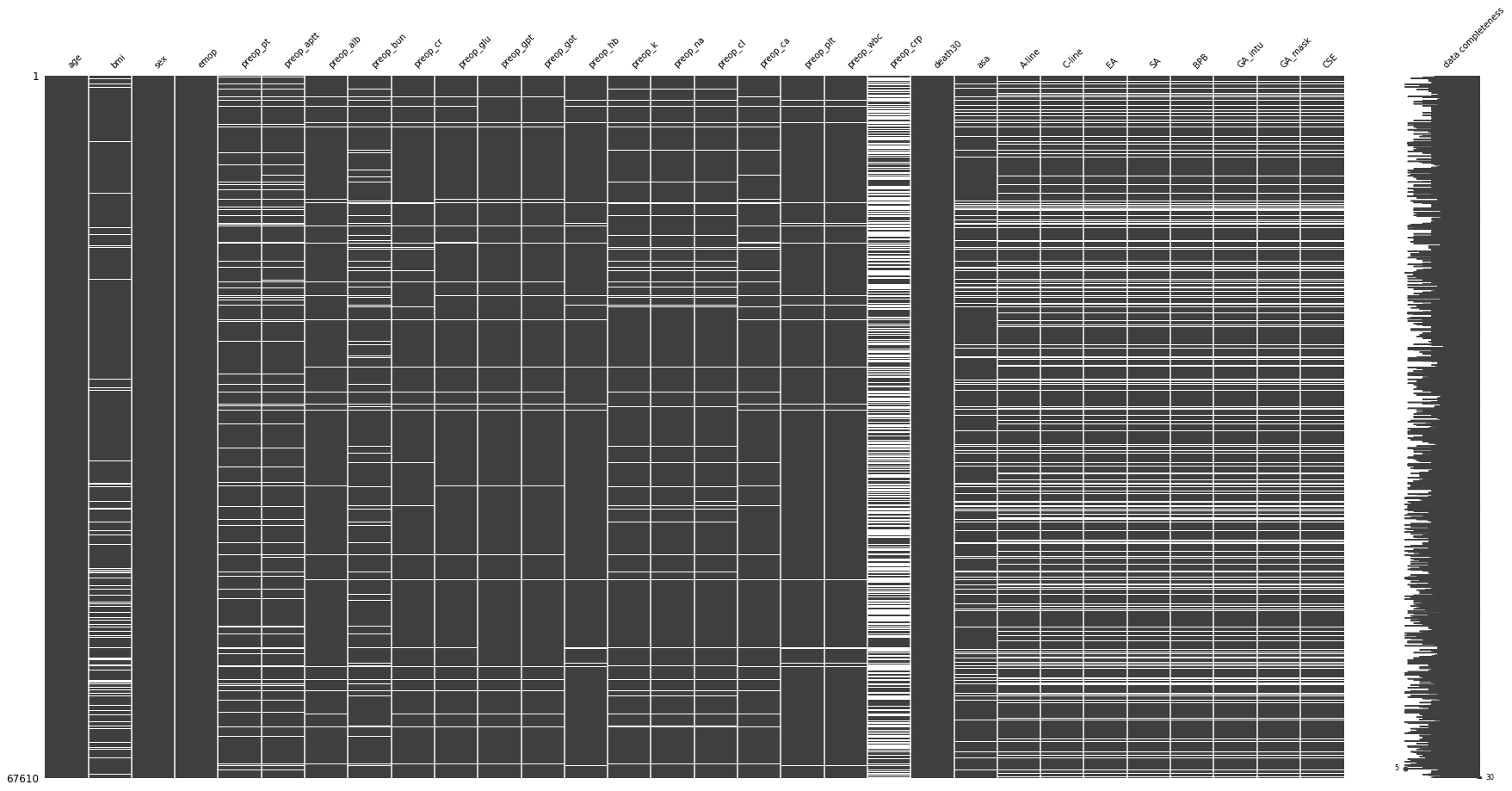


**Supplementary Figure 3.(a)** Train and validation learning curves of DNN model for arterial catheterization.

**
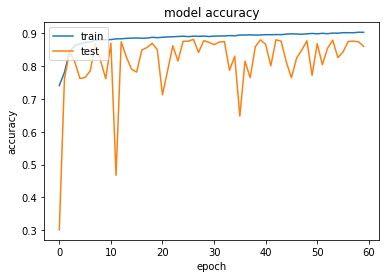

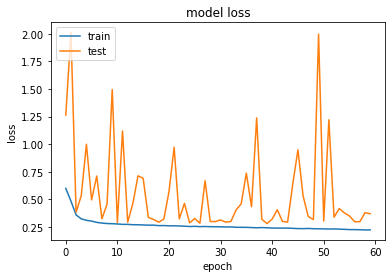
**

DNN, deep neural network.

**Supplementary Figure 3.(b)** Train and validation learning curve of DNN model for central venous catheterization.

**
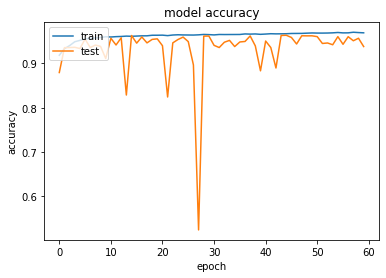

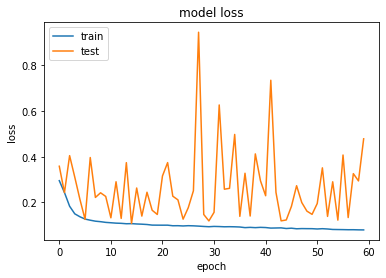
**

DNN, deep neural network.

**Supplementary Figure 4.(a)** Prediction performance of DNN model for arterial catheterization by gender using all features.


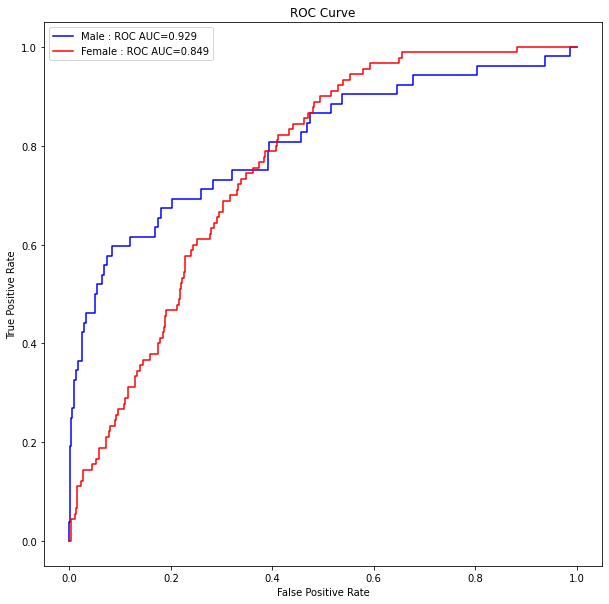

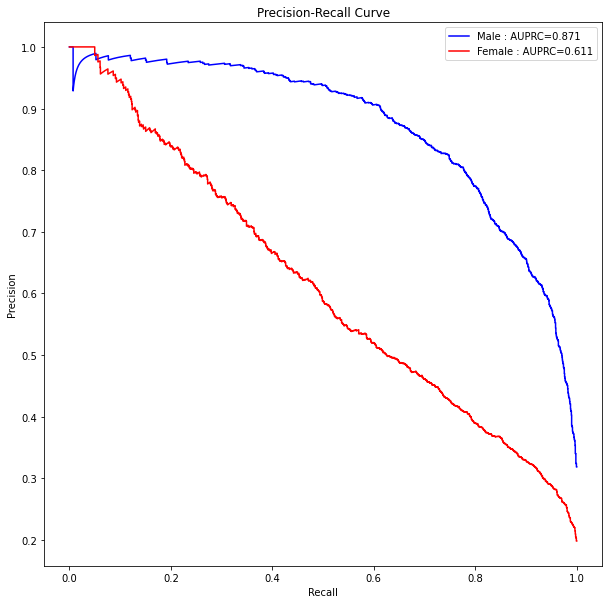


DNN, deep neural network.

**Supplementary Figure 4.(b)** Prediction performance of DNN model for arterial catheterization by age groups using all features.

**
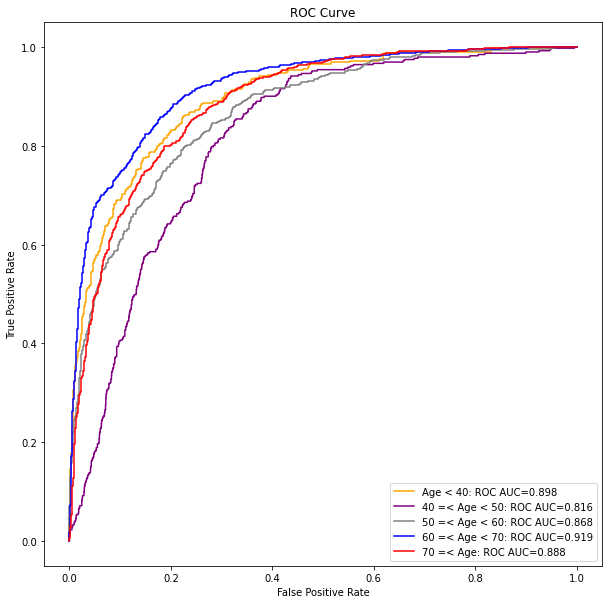

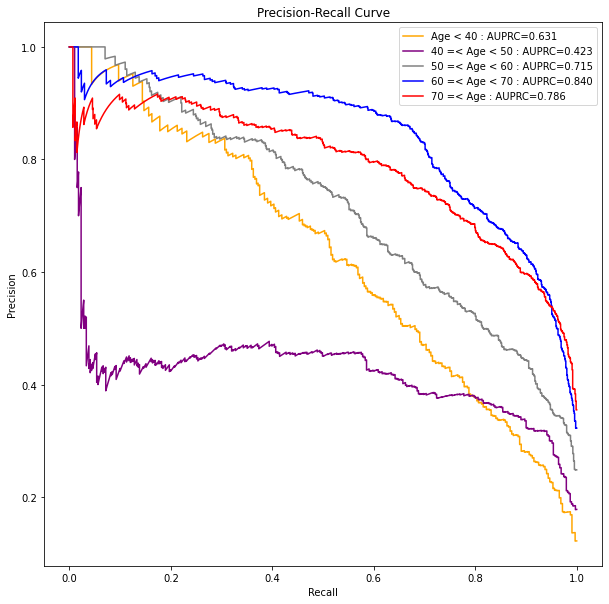
**

DNN, deep neural network.

**Supplementary Figure 4.(c)** Prediction performance of DNN model for central venous catheterization by gender using all features.


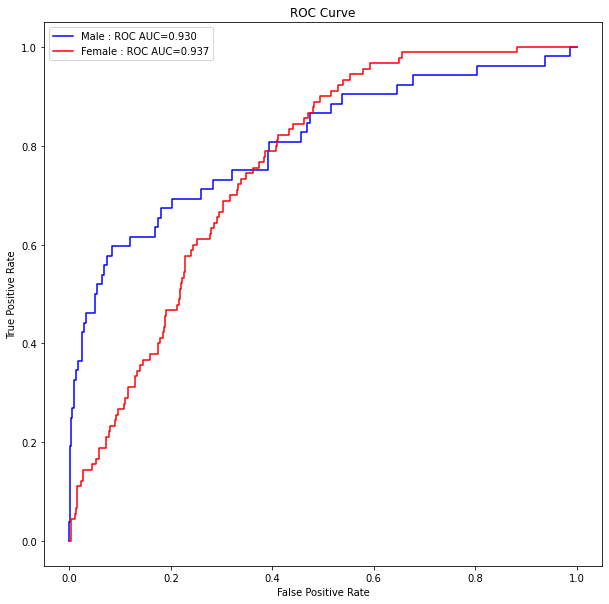

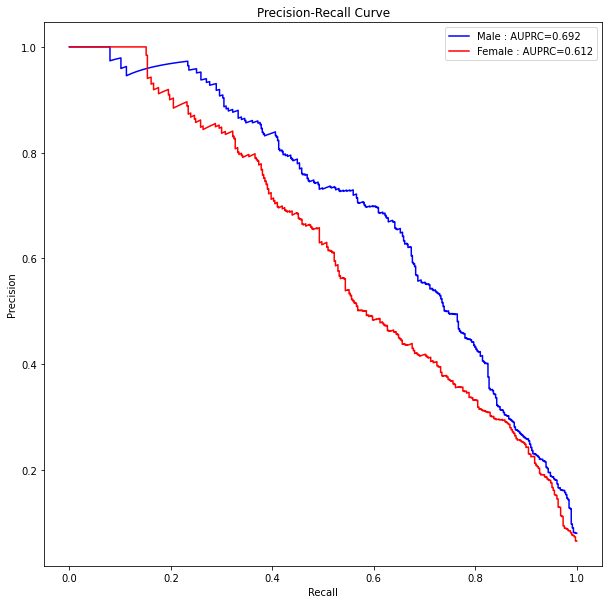


DNN, deep neural network.

**Supplementary Figure 4.(d)** Prediction performance of DNN model for central venous catheterization by age groups using all features.

**
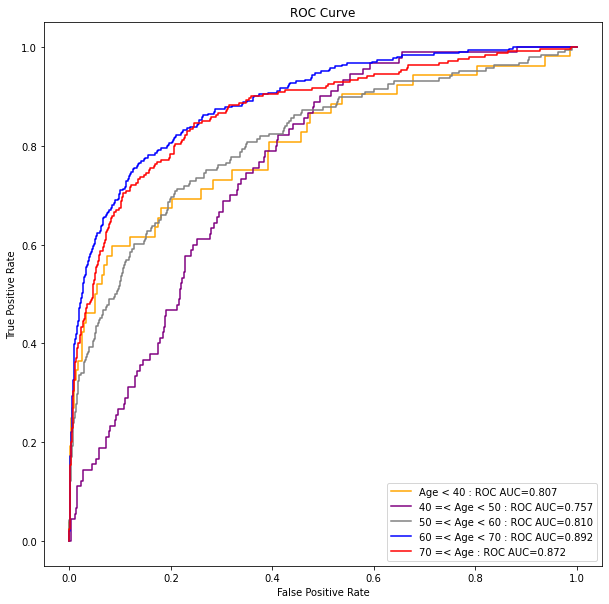

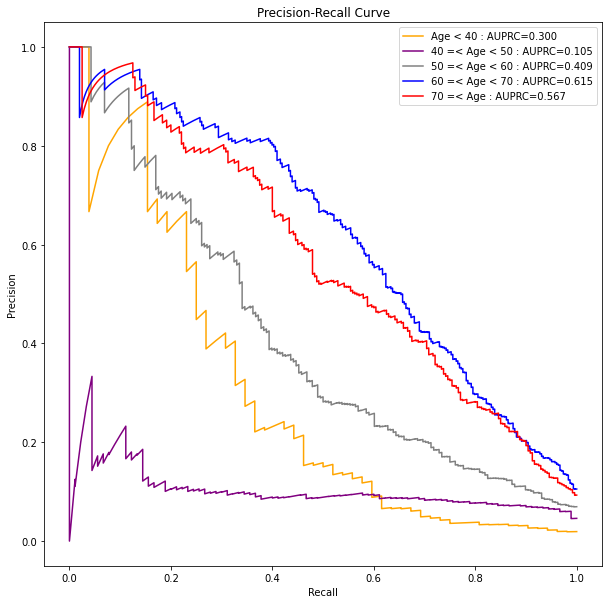
**

DNN, deep neural network.

**Supplementary Figure 5.(a)** ROC and PR curves of XGB model with 10-fold cross-validation for predictive performance of arterial catheterization using all features.


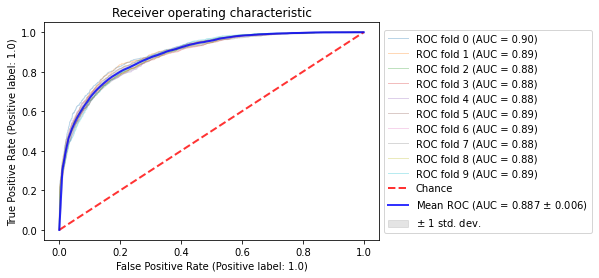


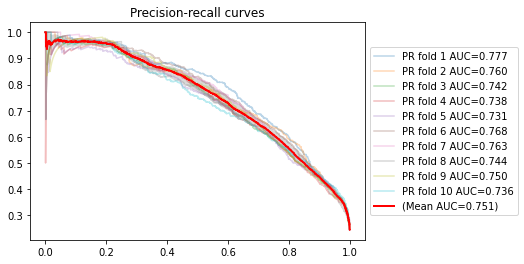


ROC, receiver operating characteristic; PR, precision-recall; XGB, extreme gradient boosting; AUC, area under curve.

**Supplementary Figure 5.(b)** ROC and PR curves of XGB model with 10-fold cross-validation for predictive performance of central venous catheterization using all features.


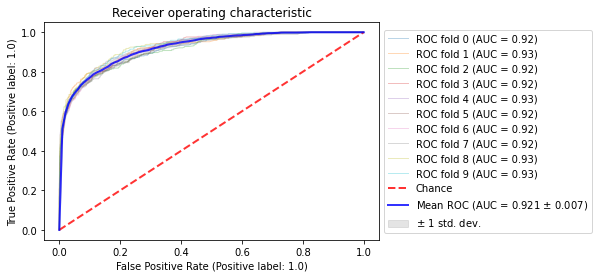

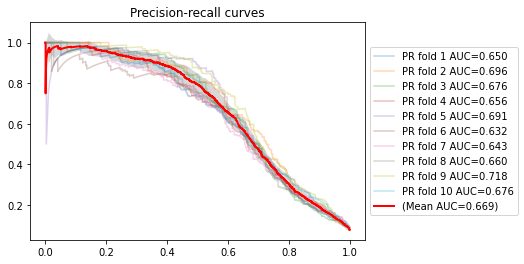


ROC, receiver operating characteristic; PR, precision-recall; XGB, extreme gradient boosting; AUC, area under curve.

**Supplementary Figure 6.(a)** Calibration curve of predictive performance for arterial catheterization according to various modeling methods using all features.


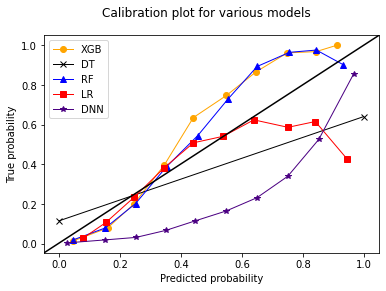


DNN, deep neural network; XGB, extreme gradient boosting; DT, decision tree; RF, random forest; LR, logistic regression.

**Supplementary Figure 6.(b)** Calibration curve of predictive performance for central venous catheterization according to various modeling methods using all features.


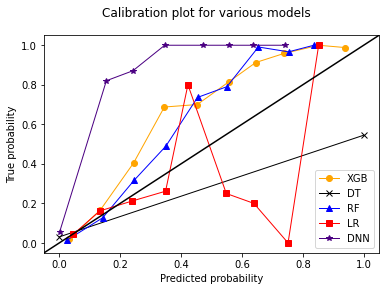


DNN, deep neural network; XGB, extreme gradient boosting; DT, decision tree; RF, random forest; LR, logistic regression.
